# A framework for a comprehensive animal welfare label: scientific, logistic, and ethical challenges

**DOI:** 10.1093/af/vfaf003

**Published:** 2025-04-22

**Authors:** Frank A M Tuyttens, Alistair B Lawrence, Siobhan Mullan

**Affiliations:** Animal Sciences Unit, Flanders Research Institute for Agriculture, Fisheries and Food (ILVO), Melle, Belgium; Department of Veterinary and Biosciences, Faculty of Veterinary Medicine, Ghent University, Merelbeke, Belgium; Animal Behaviour and Welfare Group, Scotland’s Rural College (SRUC), Edinburgh, UK; Animal Welfare Group, School of Veterinary Medicine, University College Dublin, Dublin, Ireland

**Keywords:** animal-based measures, animal welfare assessment, labeling, method of production, monitoring, resource-based measures

**Implications**

- Changes in societal expectations should drive a new framework for animal welfare labeling.
- The proposed labeling framework promotes high *potential* and *actual* animal welfare by combining resource and animal-based approaches.
- Correct uptake of this framework should effectively improve the quality of life of farmed animals.

## Introduction—A Virtuous and Comprehensive Animal Welfare Label

At least nine of the 27 EU member states have national food labeling schemes with animal welfare claims, and there is a growing plethora of private animal welfare labels (**AWL**), e.g., as part of the marketing strategy of retail companies (Lundmark et al., 2018; EC, 2022; Stygar et al., 2022). The impact of these labels on the welfare of farmed animals is generally considered disappointing (but see Mullan et al., 2016, 2022). The multitude of labels often confuse the consumer, and there seems to be a clear need for simplifying the existing state of play of these labeling schemes (BEUC, 2021; Forbes, 2021; Promarket, 2021; EC, 2022). Both the European Commission and some national authorities, such as the Flemish Government, are therefore considering options for animal welfare labeling to better transmit value through the food chain. This article wishes to contribute to these developments by proposing a framework for a virtuous and comprehensive AWL for animal products.

In our view, there can be two, nonmutually exclusive, virtuous roles of labeling food. The first role is to inform consumers (or other stakeholders) about product attributes that they, or society at large, consider relevant. This may include the country of origin, quality assurances, and the impact of the production process on the environment and climate, and on people and nonhuman animals (hereafter termed “animals”). The second role is to drive change in consumption and hence production practices. This aspiration could be to lower the environmental footprint, to improve labor condition, or—the focus of this article—to improve the welfare of animals from which these products have been derived. Recognizing that informing may also drive change and that in the process of changing consumption habits people often become more informed, it is clear that both virtuous roles of labeling may be closely intertwined.

Bearing this interconnectedness in mind, we present a novel framework for a virtuous and comprehensive AWL for animal products with the ultimate goal of improving animal welfare through changes in consumption patterns. Whilst it would be welcome if people became better informed and more aware about livestock welfare issues, ultimately we wish the effectiveness of the label to be judged by the extent that consumption (and hence production) patterns have shifted to the benefit of the animals. This contrasts with less virtuous—at least in our opinion—and sometimes hidden agendas of some AWL schemes such as sheer profit, “welfare-washing,” or the promotion of goods produced in their own country (nationalism). We would contend that the failure to truly impact animal welfare likely relates to these less virtuous agendas for AWL.

The shift we aim for may be achieved both by raising thresholds for the lower welfare products, and by a demand shift in favor of higher welfare products at the expense of lower welfare products. As higher animal welfare standards typically increase production costs (McInerney, 2004; Gocsik et al., 2016; Vissers et al., 2019; Molnár and Szőllősi, 2020), consumers will need to be encouraged not only to seek out animal-friendlier goods, but also to pay premium prices for it. Hence, to be effective, the AWL should meet some important criteria: (1) reflect animal welfare status correctly (validity), (2) be cost-efficient, and (3) align with consumer expectations, including price differentials. Indeed, lack of transparency and trust, as well as costs being too high or not being aligned with expectations (value for money) have been identified as barriers for consumers to purchase animal-friendlier products, even if they have stated in surveys that they would prefer to do so (Ingenbleek and Krampe, 2022). For voluntary and private labels, in particular, these three criteria will also largely determine the scalability, or the market uptake, of the AWL.

One possible reason for AWL not meeting consumer expectations is that they are not comprehensive, for example because they fail to cover important aspects of animal welfare (e.g., positive affective states). AWL can also fail to be comprehensive in the sense that they do not cover the entire production chain but instead focus on one particular life phase only, often the production stage on farm. A focus restricted to the on-farm phases may make farmers feel singled out whereas they have little influence on other stages of the production chain that may affect animal welfare considerably as well (e.g., genetic breeding stock, transport, and slaughter). This concern can be broadened to other aspects and activities that are often not considered by existing AWL although they are inherently linked to the entire production chain and can be associated with specific animal welfare issues, such as the killing of the nonproductive sex (e.g., male calves or chicks) or the stressful conditions of the parental stock. Hence, we include a fourth criterion for an AWL: that it is comprehensive by including all major aspects of animal welfare, all relevant parts of the production chain and the entire lifetime of the animals.

## Monitoring Animal Welfare: Challenges

At the heart of any AWL is a scheme or protocol for monitoring the welfare status of the animals concerned (Blokhuis et al., 2010). Whilst there is an EU-wide compulsory system of labeling table eggs since 2012 (based on the EU Council Directive 1999/74/EC) differentiating four poultry production methods (enriched cages, barn, free range, organic) this is not available for other species. In some cases, an industry may have agreed similar terms at a national level, for example, the Pork Provenance (i.e., an industry voluntary scheme in the UK for providing the pig meat supply chain with a set of guidelines with which to improve labeling on pork and pork products). Whilst these types of production method schemes convey some tiered information on the *potential* welfare that can be achieved they are neither comprehensive (often focusing on one life phase) nor do they assure anything about the *actual* level of animal welfare achieved. Assuming otherwise is a typical example of “animal housing reductionism” or the tendency for public (and even scientific) discourse of animal welfare, ethics, language, and labeling to be reduced to issues of housing (Scrinis et al., 2017).

Indeed, housing systems may differ in the maximal degree of animal welfare that can be realized potentially (Murphy and Legrand, 2023), but the actual welfare status can differ widely between farms with the same housing system (Sherwin et al., 2010; de Jong et al., 2022) and even farms covered by the same resource-based certification scheme (Main et al., 2003; Velarde and Dalmau, 2012; Bartlett et al., 2024). Poor welfare can occur in any production system because the actual animal welfare status is determined by complex interactions between the stockperson(s), the animals, and their environment. Since the Welfare Quality project, consensus has grown that animal welfare is, therefore, most directly assessed by so-called animal-based measures (**ABM**). Resource-based measures (**RBM**), that describe the conditions in which the animals are kept, are to be seen rather as risk and success factors that, respectively, decrease or increase the likelihood for attaining a certain level of animal welfare (Table 1). This view has since been corroborated by the European Food Safety Authority (EFSA, 2012) and animal welfare scientists increasingly emphasizing the need for animal welfare to be monitored using ABM (Main et al., 2007). This has resulted in monitoring protocols that require (trained) auditors to visit farms and assess the behavior and condition of groups of animals and/or a representative sample of individual animals (e.g., Welfare Quality®, 2009, Welfair, AssureWel). These animal-based (or welfare outcome) protocols face considerable challenges regarding logistic feasibility as well as reliability and validity of the assessments (Table 2). Moreover, it tends to be less predictable to the farmer, transporter or slaughterer whether or not ABM thresholds will be met than is the case for most RBM thresholds. This uncertainty may be a heavy burden to them.

**Table 1.** Pros and cons of assessment schemes exclusively based on resource-based welfare indicators

Pros	Cons
Commercial uptake (cost-efficient and feasible) Match with consumer expectations and perceptions Updates not needed unless housing/management changes Often quantifiable, verifiable & reliable Predictable outcome (no surprises for farmer/transporter/slaughterer) May document aspects of animal welfare hard to assess with animal-based measures (e.g., positive state, thirst, spatial needs)	Validity issues (risk factor rather than direct indicator of animal welfare; actual welfare state may vary even with the same resources) May reinforce consumer misconceptions (e.g., animal housing reductionism) No incentives to improve once criteria for certain level are met

**Table 2.** Pros and cons of assessment schemes exclusively based on animal-based welfare indicators

Pros	Cons
Validity (outcomes measures are more directly linked to animal welfare than resource-based measures) Allows comparison between housing/management systems and between animal types Implementing a continuous incentive to improve is feasible Some measures may be routinely available or can be collected (automatically) at slaughter	Focus on physical/behavioral problems (mismatch with current definitions of animal welfare that incorporate positive experiences) Communication and marketing challenges Cost-efficiency and feasibility issues (labor intensive, bio-security issues, timing, and planning of assessments) Reliability issues (sampling, observer, expectation, announcement, and time dependency effects) Unpredictable outcome (uncertainty may negatively affect the farmers’, transporters’, slaughterer’ peace of mind)

The validity of the assessment is, of course, closely linked to the concept of animal welfare and how it is defined. Being a dynamic societal concept, the definition of animal welfare has shifted during the last decennia in two major ways: (1) a stronger emphasis on the affective states as the most direct determinant of animal welfare, and (2) a greater emphasis on positive welfare in addition to indicators of welfare problems (Boissy et al., 2007; Yeates and Main, 2008; Lawrence et al., 2019). Both evolutions are seriously challenging animal-based welfare monitoring. With some exceptions such as Qualitative Behavior Assessment (Keeling et al., 2021), most ABM that are feasible and reliable tend to focus on physical health (e.g., lesions, disease, mortality, body condition) or behaviors (e.g., panting or shivering, lameness, sickness behavior) indicative of welfare issues, rather than on directly assessing emotions, let alone positive emotions (Boissy et al., 2007; Rowe and Mullan, 2022). Thus, whereas lifetime welfare status of animals is increasingly seen as the balance between positive and negative experiences (Reimert et al., 2023), current animal-based protocols largely document the negative aspects. This not only conflicts with modern views of the concept of animal welfare but also with expectations from an increasing part of society that farmed animals should not only be spared from suffering but should be granted a good life (Lawrence et al., 2018; Vigors, 2019). Accordingly, it may be expected that consumers paying premium prices for animal welfare claims increasingly expect these claims to reflect positive welfare rather than a mere reduction in welfare problems. A further problem associated with this one-sided limitation on what can be reliably and feasibly quantified using ABM is that it favors intensive production systems and disadvantages more extensive systems, as the latter provides more “psychological opportunities” (which can hardly be documented by ABM) but fewer “physical safeguards“ (which ABM focus on) (Veasy et al., 2017).

Presumably because of these feasibility, reliability, and validity issues related to animal-based monitoring, there recently appears to be a trend towards re-emphasizing the conditions in which farm animals are being kept and how they are managed. This is reflected in societal cries to ban restrictive housing systems (e.g., End the Cage Age) or certain management practices (e.g., beak trimming) and in retailers committing to demand better animal housing, management conditions, and adjusted breeding policies from their suppliers (e.g., Better Chicken Commitment). It is also reflected in the rise of so-called Method-of-Production (MoP) labels (as RBM-based proxies for AWL) by retailers (e.g., Lidl), animal welfare NGOs (e.g., Beter Leven Keurmerk in the Netherlands), as well as official authorities (e.g., the staatliche Tierhaltungskennzeichnung or the mandatory state animal husbandry label in Germany, or DEFRA’s proposed MoP label in the United Kingdom). The greater commercial uptake of these MoP labels compared to animal-based monitoring probably relates to their better cost-efficiency, feasibility, reliability, comprehensibility, controllability, predictability and by their alignment with consumer expectations (Table 1). An important downside of a MoP label—in addition to being composed of risk factors instead of direct indicators of animal welfare—is that the incentives to continue improving animal welfare once the housing and management conditions comply with a given MoP tier are limited. This is of particular concern for the more complex production systems where the real welfare status may differ a lot between and within farms (Heerkens et al., 2015; Hartcher and Jones, 2017) and tend to be lower than citizens (naively) assume or expect (Ochs et al., 2019; Alonso et al., 2020).

## A Novel Framework for a Comprehensive AWL

We therefore propose a novel framework for an impactful, virtuous, and comprehensive AWL with a fair reward and penalization system that favors high *potential* and *actual* welfare status. Our AWL consists of four tiers of increasingly better welfare that combines the best of input (resource-based) and output (animal-based) measures of animal welfare. The highest tier is labeled with three stars whereas the lowest tier has no star, and the price premium increases with the number of stars. For internal use (i.e., not communicated to the public), the lowest tier can include a separate sub-tier (“unacceptable”) if animal welfare conditions must be improved urgently before the product can be sold because they are below the legal or ethical minimum norms.

In order to align with consumer expectations and to facilitate the marketing of animal welfare, the highest animal welfare tier that can be claimed by a farmer, transporter, or slaughterer is determined by MoP requirements for the respective stages of the animal’s life. These input requirements concern the conditions in which the animals are kept, the genetics of the animals, and how they are managed from birth or hatching until slaughter. The degree to which these requirements provide opportunities for experiencing positive welfare and preventing negative welfare determines the maximum number of stars that can be achieved. The “Good Life Framework” developed by Edger et al. (2013) for laying hens and by Rowe and Mullan (2022) for broilers, beef cattle, and pigs provides a good starting point for ensuring that opportunities for the animals to experience positive welfare is included in the scheme. These increasing MoP requirements form the basis of how the different tiers are communicated to the consumers in a simple, visually attractive, and easily understandable manner (e.g., outdoor access).

As MoP requirements determine the potential rather than actual welfare status and as there are no stimuli to strive for optimal welfare once the housing and management requirements for a certain tier are met, the animals are also screened for key ABM. We propose that this screening could be conducted at the slaughterhouse to increase feasibility, comprehensiveness, and reliability (Table 3).

**Table 3.** Advantages and disadvantages of monitoring ABM in the slaughterhouse

Criterion	Explanation of pros and cons (if any)
Validity, Comprehensiveness and Reliability	*Pros* Conditions in which ABMs are recorded can be highly standardized reducing the likelihood of biases and noise due to variations in, e.g., the age or weight range of the animals, or visibility during scoring Automation can eliminate observer, expectation, announcement, and sampling biases Animals are assessed at the end of their lives, implying that indicators can be sought that give information about their welfare status not only during the on-farm stage but also during transport and slaughter *Cons* Indicators providing information on animal welfare during the on-farm and transportation phase focus on welfare problems that affect the physical appearance of the animals and fail to document other dimensions of welfare Early life welfare status is underexposed as at-slaughter ABM are less sensitive to the animals’ earlier versus recent life stages It may be difficult to determine at which life stage (on-farm, transport, slaughter) a welfare issue detected at slaughter occurred
Feasibility and Cost-efficiency	*Pros* Centralized data collection and processing: animals from multiple farms eventually end up at the slaughterhouse (no time wasted on making appointments, driving to farms, getting close to animals for observations). Data processing may include automated and immediate personalized feedback to the person responsible for each production stage (farmer, transporter, slaughterer) in the form of benchmarking and evolutions in time. No bio-security risks due to the auditing. Feasible to automate the monitoring of ABM (using various sensor technologies and artificial intelligence) and to centralize data collection and processing Animal welfare officers are already present at slaughterhouses and existing meat quality inspections can be expanded by animal welfare assessments
Consumer expectations, incl. price differential	*Pros* Given the high cost-efficiency price differentials will be minimally affected by the cost of ABM monitoring

As it is currently unrealistic to expect end of life ABM to provide an exhaustive welfare evaluation (Table 3), its main function is to capture variation of measurable (iceberg) welfare problems (e.g., tail damage in pigs or contact dermatitis in broilers) within MoP tiers. Protocols for (automated) at-slaughter monitoring of ABM have been or are being developed for several types of agricultural animals (e.g., Van Noten et al., 2022 for laying hens; Maselyne et al., 2022 for broiler chickens and pigs; Hernandez et al., 2023 for pigs; Wigham et al., 2018 for cattle). Despite these benefits and advances, further research is warranted, however, as the number and type of measures that can be used are still limited, are often focused on the physical health, and are not always well able to reflect the animals’ earlier life stages as opposed to recent issues relating to transport or slaughter (Bottacini et al., 2018; Urrea et al., 2021). For relatively long-lived animals (e.g., dairy cattle and laying hens) in particular, at-slaughter monitoring could be complemented with relevant and routinely available book keeping records such as on mortality, performance, antibiotics, and other veterinary drugs or treatments (Sandgren et al., 2009; Nyman et al., 2011; de Vries et al., 2014; Lutz et al., 2021), or with (automated sensor-based) ABM monitoring on-farm or during transportation (Rowe et al., 2021; Van Veen et al., 2023).

## How to Use and Implement the Proposed AWL Scheme?

**Figure 1.**
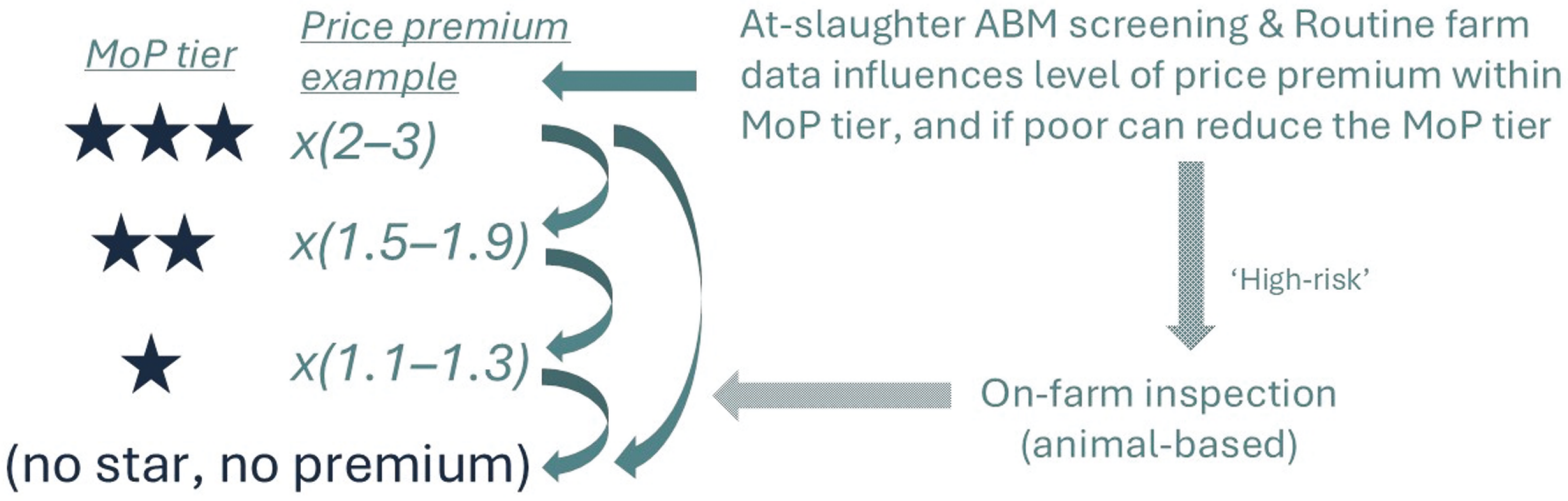
Illustration of how the at-slaughter ABM screening interacts with the four MoP tiers and associated example price premium ranges (a one, two, and three-star label allow for a premium price between respectively 10% to 30%, 50% to 90%, and 100% to 200% as compared to products without a star). ABM screening complemented with routine record data determine the exact price premium and help identify “high-risk” farms/transportations/slaughterhouses where the actual welfare seems below expectation given the welfare potential of the MoP tier. High-risk actors may lose one or more stars depending on on-site inspections by animal welfare experts. The overall ambition is to increase the market share of animal products sourced from systems with high levels of potential and actual welfare status (relative to those with a lower welfare status).

Similar to the French “Étiquette Bien-Être Animal” scheme and as recommended by Murphy and Legrand (2023), the proposed AWL consists of MoP tiers indicating increasing welfare potential and ABM monitoring to evaluate as far as possible whether the welfare potential of a given system actually translates into good welfare. The ABM screening can be used in various ways to motivate those responsible to strive for optimal welfare within their assigned MoP level. For example, each actor responsible for a production phase of a batch of animals slaughtered receives personalized feedback on the ABM in the form of benchmarking and evolution in time. This will be complemented by two financial motivators (Figure 1). Firstly, we assume that each additional MoP tier will command a price premium in the market. The ABM screening could be used to provide evidence to support a further price premium differential within a MoP tier. Suppose, for example, that those complying with the MoP requirements for 1 star are entitled to a price premium of 10% to 30%. The further price differential could entail that the exact price premium will depend on the ABM screening: it will be 10% only for those with the worst outcomes and gradually increase to 30% for those with the best outcomes within that tier. The market rational for this would be enhanced welfare assurance delivering reduced reputational risk of a poor welfare scandal on a star-labeled farm (e.g., Barnes, 2024). A clever market regulation system may be needed though to prevent a counterproductive risk that consumers and retailers would favor the lowest welfare suppliers within a tier as these would be the cheapest. Secondly, (repeatedly) poor ABM outcomes could result in loss of stars (and thus in a lower price premium range). A transparent decision tree would determine the conditions under which decisions to reduce stars can take place and how. Before deciding to reduce the number of stars one may first give a warning and allow the actor to take corrective actions to improve the welfare problems. Alternatively, and if resources allow, one may decide that an inspection of the farm, transportation company or slaughterhouse by a (certified) welfare expert is needed to determine whether a loss of stars is justified. We would not advise that stars can be gained by excellent ABM outcomes because at-slaughter ABM screening is currently too limited to be used as a comprehensive assessment of actual animal welfare level of all production phases and because it would complicate communication with consumers who associate the number of stars mainly with MoP criteria.

For maximum transparency and impact, it is advisable that all animal-derived products are labeled and not only the higher welfare ones. Indeed for increasing consumer awareness and for reducing welfare washing opportunities, it is important that also the lowest welfare tier is clearly visible and recognized as such to the consumer. The AWL scheme would be managed transparently and preferentially by an international authority (e.g., EU-commission), alliance (e.g., Global Animal Welfare Assurance: Rowe et al., 2021) or animal welfare NGO, rather than a national authority or private food company or retailer which tend to be trusted less by citizens (EC, 2022). Furthermore, the more widespread the label is used the greater its potential to reduce confusion among consumers about the meaning of multiple labels and to incorporate it in international trade of products from animals reared to a definable welfare status. Existing assurance schemes and food businesses have expressed an interest in greater global standardization or recognition of equivalence and authenticity of higher welfare claims (Rowe et al., 2021). Established, accredited, and independent professional farm assurance scheme organizations could be contracted to conduct the actual verification of animal welfare status using the criteria of the proposed AWL scheme.

The (international) organization that manages the AWL may allow negotiation on specific country or user differences, and use it as a standard against which other labels can be calibrated or benchmarked. Moreover, they could even draft best practice guides on how retailers and governments could make optimal use of the AWL for improving farm animal welfare, and categorize them on how well they are able to do so. For example, retailers that wish to be certified as “animal friendly” may be required to exclusively sell animal products with one (or more) welfare stars, to actively promote the highest welfare levels, or to make the least profit margin from products with the highest welfare. Similarly, (local) authorities that wish to be certified for animal-friendly governance may be required to finance and promote the uptake and visibility of the AWL, to promote products with the highest number of welfare stars at the expense of the products with fewer or no stars, and to finance studies to evaluate and improve the impact of the labeling scheme on actual animal welfare. Albeit an aspiration that may be hard to realize, governments may consider introducing a tax on purchase of products of low welfare (or high environmental) burden in order to finance the labeling scheme and these affiliated requirements. Model simulations have shown that such a tax can have a strong steering effect (Edjabou et al., 2013; Bonnet et al., 2018; Dogbe and Gil, 2018; Moberg et al., 2021; Funke et al., 2022; Roosen et al., 2022). A referendum choice experiment with German citizens, revealed that support for a tax on meat is stronger if justified by animal welfare rather than climate change mitigation (Perino and Schwickert, 2023).

## Conclusion

By combining RBM and ABM, we propose a framework for a comprehensive and tiered AWL with the primary virtuous goal of improving the quality of life of farmed animals as effectively as possible. MoP requirements for the various stages of the animals’ lives determine the highest potential welfare tier that can be obtained by a farmer, transporter, or slaughterer. To motivate these actors to continuously strive for optimal actual welfare status, the animals are also screened for key ABM. For reasons of cost-efficiency, comprehensiveness, reliability, and potential for automation this monitoring is conducted at-slaughter (but can be complemented with relevant routine record data). The actors are given feedback about these measures and poor scores may result in a lowered price premium, a warning, an in situ inspection, and ultimately a lowering of the welfare label tier. This labeling framework shows most promise for meeting our requirements regarding validity, cost-efficiency, alignment with consumer expectations and comprehensiveness. The main scientific challenges include: (a) the compilation of RBM per welfare tier that correctly reflects increasing potential for positive experiences and decreasing potential for negative experiences for each production phase and each type of farm animal; (b) the development and validation of automated at-slaughter ABM that are validated against actual welfare status during the various production stages. Given the remarkable progress in animal welfare science, alongside advances in technology and artificial intelligence during the last decade we believe that both challenges can be addressed successfully within a relatively short time-frame. A major marketing challenge is that consumers ought to consider the price differentials for better welfare products as good value for money. Moral challenges might include that lowering the animal welfare footprint often increases the environmental footprint, and that any type of human exploitation of other sentient beings is met with increasing ethical concerns especially if the animals’ overall quality of life remains below neutral.

## About the Authors

**Frank A.M. Tuyttens** (BSc Hons Biology, University of Edinburgh; D.Phil in Zoology, University of Oxford) is head of the Farm Animal Welfare & Behaviour Research Group at ILVO (Belgium), visiting professor at the Faculty of Veterinary Medicine (Ghent University, Belgium) and member of EFSA’s Animal Health & Animal Welfare (EFSA-AHAW) panel. He is also a member of the steering committee of the Flemish Council for Animal Welfare and chair of the scientific advice committee of the “Beter voor Dieren” animal welfare label being developed in Flanders. His research interests focus on assessing and improving the quality of life of farm animals. **Corresponding author:**frank.tuyttens@ilvo.vlaanderen.be

**Alistair B. Lawrence** (BSc Zoology, University of St Andrews; PhD in Applied Animal Behaviour, University of Edinburgh) is a Professor of Animal Behaviour and Welfare at SRUC and the University of Edinburgh. He has studied a diverse range of subjects, including the causes of abnormal behaviors in confined animals, the welfare of farrowing (parturient) sows, and the roles of prenatal factors and genetics in health and welfare. More recently, he has returned to a long-standing interest in positive animal welfare, which he studies conceptually and experimentally using pigs and laboratory rats as models. In addition to researching biological aspects of animal welfare, Alistair has integrated his research with various forms of social science, including economics. His interest in labeling of animal welfare products emerges from the difficulty in resetting markets to pay a fair price for animals to live “good lives.”

**Siobhan Mullan** is a Professor of Animal Welfare and Veterinary Ethics at University College Dublin. She has a veterinary background, and her research interests focus on utilizing welfare assessments to drive large-scale animal welfare improvements, promoting positive welfare experiences for animals, and the policy intersection between animal welfare and other societally desirable ethical elements such as environmental sustainability.
